# Applications of Anterior Segment Optical Coherence Tomography in Cornea and Ocular Surface Diseases

**DOI:** 10.1155/2016/4971572

**Published:** 2016-09-19

**Authors:** Sang Beom Han, Yu-Chi Liu, Karim Mohamed Noriega, Jodhbir S. Mehta

**Affiliations:** ^1^Department of Ophthalmology, Kangwon National University Hospital, Seoul, Republic of Korea; ^2^Singapore National Eye Centre, Singapore; ^3^Singapore Eye Research Institute, Singapore; ^4^Department of Ophthalmology, Yong Loo Lin School of Medicine, National University of Singapore, Singapore; ^5^Department of Ophthalmology, Autonomous University of Nuevo Leon, Monterrey, NL, Mexico

## Abstract

Optical coherence tomography (OCT) is a noncontact technology that produces high-resolution cross-sectional images of ocular tissues. Anterior segment OCT (AS-OCT) enables the precise visualization of anterior segment structure; thus, it can be used in various corneal and ocular surface disorders. In this review, the authors will discuss the application of AS-OCT for diagnosis and management of various corneal and ocular surface disorders. Use of AS-OCT for anterior segment surgery and postoperative management will also be discussed. In addition, application of the device for research using human data and animal models will be introduced.

## 1. Introduction

Optical coherence tomography (OCT), first developed by Huang et al. [1], is a noncontact imaging technology that produces detailed cross-sectional images (tomography) using low-coherence interferometry in biological tissues [1]. The use of anterior segment OCT (AS-OCT) imaging was first introduced in 1994 [2], and AS-OCT became commercially available in 2001 [3]. With dramatic development of imaging techniques, AS-OCT is currently used for analysis of anterior segment structures worldwide.

In this review, we aimed to provide information on the clinical and research applications of AS-OCT in corneal and ocular surface disorders.

## 2. Principles and Devices

The cross-sectional imaging capability of OCT is analogous to ultrasound biomicroscopy (UBM), but OCT has differences as follows: (1) It requires no contact, which prevents patient discomfort and image distortion. (2) Higher resolution enables detailed analysis of fine anatomic structures. (3) OCT uses infrared light instead of sound, although its principle is similar to that of ultrasound or radar imaging [4].

Based on low-coherence interferometry, OCT compares the time-delay and intensity of infrared light reflected from the tissue structures against a reference beam [5], and this interference pattern leads to a cross-sectional image of the tissue of interest [4]. To obtain an OCT image, multiple scans are performed at a series of lateral locations to create a series of axial scans (A-scans), and these A-scans are combined into a composite two-dimensional image, a B-scan, or cross-sectional tomography [4, 6].

Currently, two different OCT platforms are currently available: time domain (TD-OCT) and spectral domain (SD-OCT). In TD-OCT, cross-sectional images are produced by varying the position of the reference mirror [4]. The TD-OCT includes Visante OCT (Carl Zeiss Meditec, Oberkochen, Germany) and slit-lamp OCT (Heidelberg Engineering GmbH, Heidelberg, Germany) [7]. Both devices use a longer wavelength (1310 nm) light compared with that used for retinal imaging (830 nm), which result in reduced scattering and less signal loss in opaque media [3]. This allows deeper penetration through sclera and limbus for the visualization of the scleral spur and iridocorneal angle, with a 15–20 *μ*m resolution [8]. Another advantage of TD-OCT is a wider area of capture in a single image, whereas SD-OCT processes only a small component of the anterior segment [4, 6]. Visante OCT has 16 mm scan width and almost 6 mm scan depth in tissue, which are sufficient for anterior chamber biometry [9].

SD-OCT, also known as Fourier-domain OCT (FD-OCT), uses a stationary reference mirror [4, 10]. The interference between the sample and reference reflection is detected as a spectrum [4, 10]. A spectrometer is used to detect the signal by varying the wavelength of the light source with time [11]. Mathematical calculation using Fourier transformation of the spectral interferogram produces the cross-sectional images [4, 10].

SD-OCT devices include the Spectralis (Heidelberg Engineering GmbH, Heidelberg, Germany), RTVue (Optovue, Inc., CA, USA), and Cirrus OCT (Carl Zeiss Meditec, Oberkochen, Germany) [7]. In TD-OCT, the speed of image capture is limited by the velocity of mechanical movement of the reference mirror. By contrast, SD-OCT is freed from the limitations of a moving reference mirror [12], and the source A-scans are all captured simultaneously, leading to 10 to 100 times higher speed of image acquisition [4]. By employing shorter wavelengths (830 nm) compared to TD-OCT, SD-OCT devices are capable of improved axial resolutions of 4–7 *μ*m [12, 13]. However, SD-OCT devices have a disadvantage that horizontal scan width is limited to 3–6 mm and scan depth is shorter compared to TD-OCT [5]. Swept-source OCT (SS-OCT), a slightly different form of SD-OCT, such as Casia SS-1000 OCT (Tomey, Nagoya, Japan), uses a light with a 1310 nm wavelength and horizontal scan width of 16 mm but carried an axial resolution of only 10 *μ*m [14].

Ultrahigh-resolution OCT (UHR-OCT) is capable of axial resolution of 1–4 *μ*m, with scan width of 5–12 mm [5, 15–17]. Improved axial resolution was attained by using a light source with a broad bandwidth of more than 100 *μ*m and a spectrometer that can detect the fringes reflected from both reference and sample arms [5].

Development of UHR-OCT enables the precise imaging of the individual corneal and conjunctiva layers [4, 5, 15, 18, 19], tear film and meniscus [16, 17], and contact lens interfaces [16, 17, 20–22]. UHR-OCT can also be used for differentiation among various corneal and ocular surface pathologies, including ocular surface squamous neoplasia (OSSN), lymphoma, pterygium, melanosis, and Salzmann nodular degeneration [23–26]. Although most articles regarding UHR-OCT are based on the data from custom-built machines, commercially available UHR-OCT devices exist, that is, the Bioptigen Envisu (Bioptigen Inc., Research Triangle Park, NC, USA) and the SOCT Copernicus HR (Optopol Technologies SA, Zawiercie, Poland) [27].

## 3. AS-OCT for Diagnosis of Corneal and Anterior Segment Diseases

### 3.1. Conjunctival Diseases

AS-OCT is a reliable tool for measurement of the cross-sectional area of conjunctiva prolapsing into the tear meniscus in patients with conjunctivochalasis, which can also evaluate the effect of thermoreduction therapy [28].

In pterygium and pinguecula, SD-OCT shows subepithelial hyperreflective wedge-shaped mass with a thin overlying epithelium [29, 30]. In UHR-OCT, thin or normal epithelium overlying dense, hyperreflective, subepithelial lesion is observed [27]. In pseudopterygium, SD-OCT images demonstrate that the overgrowing membrane is not really attached to the underlying cornea [30].

In conjunctival lymphoma, a hyporeflective subepithelial lesion with a thin, slightly hyperreflective layer of uninvolved subepithelial around the lesion is observed in SD-OCT images [29].

In melanoma or nevi, SD-OCT displays epithelial hyperreflectivity, most intense in the basal layer with slight hyperreflectivity throughout the epithelium with discrete subepithelial lesions [29]. UHR-OCT shows normal thickness or slightly thicker overlying epithelium with variable hyperreflectivity [27]. Intralesional cystic space can be observed in nevi, although this does not exclude malignancy [23, 26]. Intense shadowing of sublesional tissue is visible in most melanomas [27, 29]. Thick lesions may exhibit significant optical shadowing of deeper structures due to pigment in the mass [27, 31].

### 3.2. Anterior Segment Tumors

In OSSN, SD-OCT and UHR-OCT show hyperreflective, thickened epithelium with abrupt transitions from normal to abnormal epithelium [27, 29]. UHR-OCT can also be used for the monitoring of treatment response in OSSN [27]. With resolution of OSSN, UHR-OCT images demonstrate progression toward epithelial normalization: reduced thickness and hyperreflectivity with less distinct transition zone [27]. Complete resolution of OSSN can be confirmed by UHR-OCT, which shows normalization of epithelial appearance [27].

In Salzmann nodular degeneration, SD-OCT and UHR-OCT images display dense, hyperreflective subepithelial lesion overlying Bowman's layer with normal surrounding epithelium [27, 29].

In most iris and ciliary tumors, UBM has superior ability to penetrate large or highly pigmented iris tumors and ciliary body tumors; thus, it can provide superior imaging quality and reproducible measurements of the lesions [32, 33]. However, as a noncontact technique, SD-OCT can be a reliable alternative in small nonpigmented anterior iris tumors [32, 34].

### 3.3. Corneal Diseases

**Figure 1:**
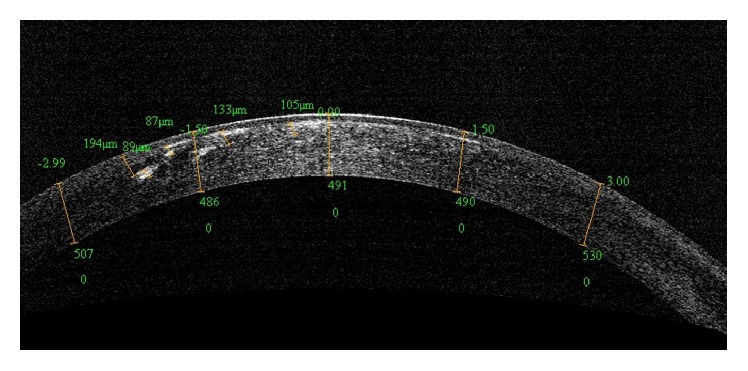
An AS-OCT image of a patient with granular dystrophy. AS-OCT can accurately show the location and depth of the visually significant stromal haze.

SD-OCT is an accurate method of predicting the depth of phototherapeutic keratectomy required to remove visually significant stromal haze in patients with granular corneal dystrophy (Figure 1) [35]. In patients with Fuchs' dystrophy, UHR-OCT is a valuable tool for* in vivo* imaging of Descemet's membrane (DM) [15]. UHR-OCT showed that DM in patients with Fuchs' dystrophy appears as a thickened band of 2 opaque lines: smooth anterior line and wavy and irregular posterior line with areas of localized thickening [15]. The DM thickness measured using UHR-OCT also correlated significantly with that measured by light microscopy [15].

In patients with microbial keratitis, corneal infiltration imaged as a hyperreflective area in the corneal stroma, retrocorneal pathologic feature, and AC inflammatory cells can be observed using AS-OCT [36]. Serial AS-OCT evaluation with measurement of infiltrate thickness and corneal thickness can be a tool for monitoring of treatment response [36]. Fuentes et al. [37] investigated the cornea of the patients with keratoconus using SD-OCT and showed that increased epithelial thickness, stromal thinning at the cone, anterior hyperreflection at the Bowman's layer, and the absence of stromal scarring are associated with increased risk of corneal hydrops [37]. AS-OCT can be helpful for the visualization of cornea and anterior chamber (AC) in cases of acute hydrops. Vanathi et al. [38] used AS-OCT for evaluation and monitoring of acute hydrops in a patient with pellucid marginal corneal degeneration, in which AS-OCT visualized intrastromal clefts with DM detachment and their resolution after treatment with intracameral SF_6_ [38]. AS-OCT can generate consistent pachymetry mapping in the presence of corneal scars [39]. It is also useful for the measurement of scar depth [39].

AC inflammation can also be evaluated using AS-OCT. Igbre et al. [40] showed that AS-OCT is a useful technique for grading AC cells [40]. In particular, AS-OCT can be used as an imaging modality in detecting AC inflammation in uveitis in eyes with decreased corneal clarity and compromised AC visualization due to corneal edema [41].

### 3.4. Dry Eye Disease

Studies have shown that AS-OCT is a reliable tool for quantitative evaluation of tear film thickness and tear film meniscus, suggesting the possibility of AS-OCT as a tool of diagnosis and follow-up of dry eye disease (DED) [42–46]. The remarkable finding is that AS-OCT findings showed good agreement with patient-reported subjective symptoms [44]. Qiu et al. [47] reported the diagnostic accuracy of AS-OCT in patients with Sjögren syndrome is higher than that in those with aqueous or lipid tear deficiency.

AS-OCT can also be an innovative clinical tool for monitoring of treatment responses in DED. Ibrahim et al. [48] revealed that measurement of tear meniscus height using AS-OCT might be effective in monitoring tear meniscus changes after punctal occlusion. Bujak et al. [49] also demonstrated the sequential changes of tear meniscus after artificial tear instillation with SD-OCT.

Recently, Hwang et al. [50] introduced a method of producing three-dimensional images of meibomian glands by reconstructing tomograms of these glands captured with high speed SD-OCT, suggesting that AS-OCT also can be used in meibomian gland disorders.

### 3.5. Anterior Segment Trauma

AS-OCT can be a useful tool in ocular injuries [10]. Its high resolution is optimal for evaluating depth of injury to the cornea or sclera and the type, size, and location of the foreign body [51, 52]. Noncontact scanning capability is also ideal for prevention of further tissue damage in fragile eyes and reducing discomfort of patients. It can also adequately assess the extent and depth of corneoscleral injuries in the setting of media opacity, which is critical for the diagnosis and follow-up of corneoscleral injuries, that is, monitoring of corneal healing process after amniotic membrane transplantation for a corneal burn [10, 51].

### 3.6. En Face OCT

En face OCT can visualize ocular surface changes that are not detectable using conventional B-scan OCT in patients with corneal dystrophies, keratitis, pterygium, conjunctivochalasis, or OSSN [53]. Although its resolution is lower than that of* in vivo* confocal microscopy, it allows overall visualization of the lesions due to the larger scan width [53]. Compared with* in vivo* confocal microscopy, en face OCT is also advantageous because it is a noncontact method that allows easy and rapid image capture [53]. 

## 4. Use of AS-OCT in Anterior Segment Surgery

### 4.1. AS-OCT in Cataract Surgery

Use of AS-OCT for preoperative planning includes calculation of intraocular lens (IOL) power, evaluation of lens, AC, and angle structures, and assessment of risk factors for postoperative complications [54]. Wong et al. [55] demonstrated that lens density measurement using AS-OCT was reliable and correlated with the Lens Opacity Classification System Version III grading scores. Measurement of corneal power using SD-OCT showed high repeatability and reproducibility [56]. In particular, AS-OCT can be an innovative tool for measurement of the corneal power for IOL power calculation in patients with prior keratorefractive surgery [57]. Tang et al. [57] showed that IOL power calculation based on SD-OCT data in patients with previous myopic laser vision correction has equal or better predictive accuracy compared with current standards.

Successful intraoperative use of AS-OCT has been described for* in vivo* assessment of clear cornea wound architecture and OCT-guided femtosecond laser-assisted cataract surgery [54]. Das et al. [58] recently showed that microscope integrated intraoperative real-time OCT using the RESCAN 700 (Carl Zeiss Meditec, Oberkochen, Germany) was helpful during all the critical steps of cataract surgery, that is, evaluation of corneal wound architecture, position of IOL, wound gaping at the end of surgery, and the adequacy of stromal hydration [58]. It was also helpful for determining the adequate depth of trenching and differentiation of true posterior polar cataracts from suspected cases intraoperatively [58]. Most commercially available laser systems, such as LenSx (Alcon LenSx Lasers Inc., Aliso Viejo, CA, USA), Catalys (Optimedica, Sunnyvale, CA USA), and VICTUS (Technolas Perfect Vision GmbH, Munich, Germany), utilize SD-OCT for three-dimensional high-resolution reconstruction of the anterior segment structures to improve safety and precision of laser cataract surgery [54].

**Figure 2:**
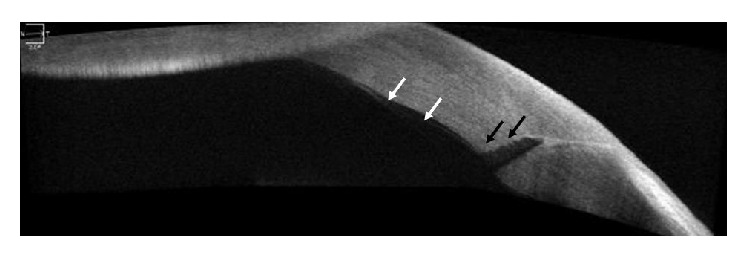
A TD AS-OCT image demonstrating localized Descemet's membrane detachment (white arrow) adjacent to the wound and internal wound gaping (black arrow) that was not detected in slit-lamp examination.

AS-OCT is also used in postoperative management after cataract surgery. AS-OCT is a reliable option for evaluation of corneal incisions after cataract surgery [10, 58–61]. It can be used for the assessment of corneal epithelial remodeling following cataract surgery [62]. It is also useful in the detection of postoperative gape of small-incision clear cornea wounds or localized DM detachment in the immediate postoperative period that cannot be observed with slit-lamp microscopy (Figure 2) [63]. AS-OCT is also useful for evaluation of the location and stability of IOL [64]. Use of AS-OCT for the diagnosis of capsular block syndrome after cataract surgery was also introduced [65].

In addition, AS-OCT is reliable tool for measurement of AC depth, cornea to IOL distances, IOL to crystalline lens distance, and iridocorneal angles in patients with phakic IOL [9, 66].

### 4.2. Corneal Transplantation

**Figure 3:**
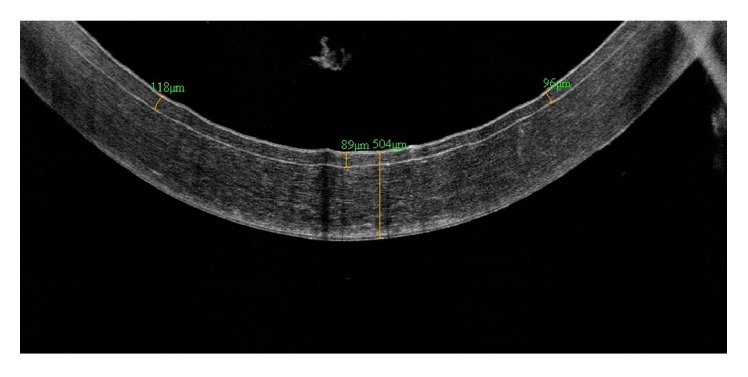
An AS-OCT image of precut donor cornea for Descemet's stripping automated endothelial keratoplasty (DSAEK) taken for evaluation of thickness of graft donor tissue.

AS-OCT is a valuable tool in corneal transplantation surgery, particularly in lamellar transplantation [10]. Preoperatively, AS-OCT can be used for evaluation of graft donor tissue for thickness and tissue preservation (Figure 3) [67, 68].

**Figure 4:**
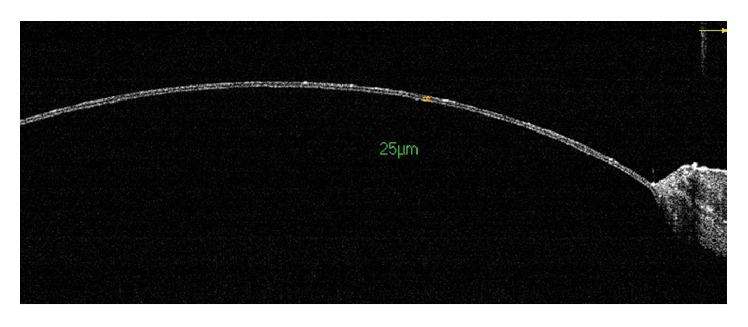
An intraoperative AS-OCT image taken during deep anterior lamellar keratoplasty (DALK) that confirms an optimal descemetic or predescemetic dissection.

**Figure 5:**
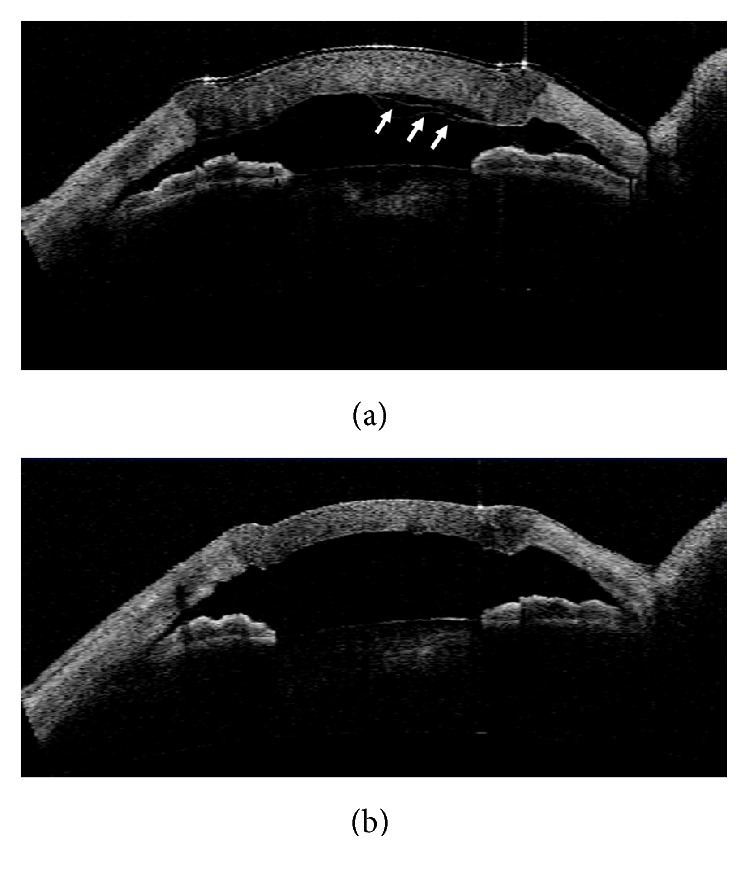
AS-OCT images demonstrating detachment of the Descemet's membrane (DM; white arrow) after DALK (a) and reattachment of the DM after intracameral air injection (b).

In deep anterior lamellar keratoplasty (DALK), intraoperative AS-OCT helps the surgeon's decision making in several steps of the surgery and to achieve an optimal descemetic or predescemetic dissection (Figure 4) [69]. When attempting big-bubble technique, AS-OCT images allow precise evaluation of the depth reached by the cannula tip used for pneumatic dissection and can confirm the decision to proceed with air injection, which may improve the success rate of big-bubble formation [70]. Lim et al. [71] demonstrated that AS-OCT can provide valuable information on donor apposition and DM detachment after DALK (Figure 5). They also showed that AS-OCT is useful for visualization of the complications of Descemet's stripping automated endothelial keratoplasty (DSAEK), such as graft dislocation, primary graft failure, AC crowding with consequent chamber angle encroachment, and pupillary block [71]. Hand-held AS-OCT can be an innovative tool for assessment of the host-donor interface in DASEK [72]. After DSAEK or Descemet's membrane endothelial keratoplasty (DMEK), DM detachment is often difficult to detect with slit-lamp evaluation in cases of persistent corneal edema. In this situation, AS-OCT can be a useful option for visualization of graft detachment [73–76]. Yeh et al. [77] revealed that AS-OCT scan at 1 hour after DMEK showed the best predictive value on 6-month graft adherence status. Shih et al. [78] also demonstrated that corneal thickness measured with AS-OCT at 1 week postoperatively was an important predictor of DSAEK failure [78]. Both studies suggest that AS-OCT findings at early postoperative period may be helpful for decision making on reintervention after posterior lamellar keratoplasty [77, 78]. AS-OCT is also able to show the presence of residual DM in the recipient cornea in a patient with a failed DSAEK, supporting the assumption that inadequate DM stripping may be the cause of graft failure [79].

In patients with Keratoprosthesis (KPro) implantation, there is no standardized method for evaluation of the implanted KPro and adjacent tissues [80]. Due to limited visualization, it is often difficult to detect serious complications such as retroprosthetic membrane, wound gape, and angle closure. In these cases, AS-OCT may play an important role. AS-OCT can show the presence of retrokeratoprosthetic membranes and a gape in the interface and lack of epithelial sealing around the KPro edge [81, 82]. Qian et al. [80] also suggested that AS-OCT can demonstrate anatomic changes including angle closure, peripheral anterior synechiae, iris-KPro backplate touch, and graft-host interface changes that cannot be detected otherwise.

### 4.3. Refractive Surgery

SD-OCT and UHR-OCT enable the precise measurement laser* in situ* keratomileusis (LASIK) flap thickness and the residual stromal bed thickness before LASIK enhancement to avoid a post-LASIK ectasia [83, 84].

Intraoperative examination using hand-held SD-OCT system (Bioptigen Inc., Morrisville, NC, USA) allows more accurate evaluation of the flap characteristic before flap edema and stromal bed hydration changes accuracy [85]. SD-OCT is also used for evaluation of the flap and stromal bed after femtosecond lenticule extraction [86].

AS-OCT is also useful for the diagnosis and management of the complications after keratorefractive surgery. Interface fluid syndrome (IFS) is a flap-related complication of LASIK surgery characterized interface haze, fluid collection, and flap edema [87]. Han et al. [87] reported that SD-OCT is a valuable tool for visualization of these findings and can also be used for confirmation of resolution of interface fluid collection and haze after treatment. SD-OCT is also used for quantitative assessment of the infiltration in the eye with post-LASIK corneal inflammation [88].

### 4.4. Surgeries for Keratoconus and Keratectasia

Efficacy of AS-OCT in the management of keratoconus and postoperative keratectasia has also been reported. AS-OCT allows the determination of the precise depth and position of the intrastromal corneal ring segments when placing the implants in patients with keratoconus and helps to avoid depth-related complications including epithelial-stromal breakdown or perforation into the AC [4, 6]. Corneal collagen crosslinking (CXL) is also used for the stabilization of the corneal stroma by increasing its rigidity in patients with keratoconus or postoperative keratectasia [6]. During the CXL procedure, AS-OCT can localize the demarcation line that indicates the transition zone between the treated and the untreated stromal tissue and measure the depth of the line [6]. AS-OCT can also be used for the evaluation of changes in corneal epithelial and stromal thickness after CXL [89].

## 5. Assessment of Anterior Segment Biometry

OCT does not require direct probe contact to the eye or water immersion; thus, it eliminates the risk of image distortion and provides higher resolution images than UBM [90]. Studies demonstrated that AS-OCT is more accurate and repeatable compared with UBM or slit-lamp photography in anterior segment biometry [4, 8, 91, 92].

AS-OCT can generate various pachymetric maps of the cornea, which is helpful for detecting keratoconus or keratectasia after refractive surgery [9]. Because it does not require contact lens removal, it is an ideal tool for monitoring of corneal thickness changes caused by contact lens wear [93]. Temstet et al. [94] reported that the thickness and location of the thinnest corneal zone determined by the SD-OCT epithelial mapping might be useful for the early diagnosis of* Forme fruste keratoconus*. In a study by Rocha et al. [95], SD-OCT demonstrated significant differences in central and regional epithelial thickness profile between keratoconus, ectasia, and normal eyes, with significant variability and unpredictability in ectatic eyes. Development of UHR-OCT also enables the evaluation of each layer of the cornea [96].

Evaluation of the AC dimensions, including the AC depth [97], AC angle [7], the angle-to-angle width [4], or the lens thickness and density, is also enabled with the development of AS-OCT [55].

Changes of the biometry of anterior segment dimensions during accommodation can also be evaluated using AS-OCT [98]. Using extended scan depth OCT, Li et al. [98] demonstrated that pupil diameter, AC depth, and anterior and posterior surface curvatures of lens became significantly smaller during accommodation, and lens thickness significantly increased with accommodation. Decrease in the anterior and posterior surface curvatures were also found in another study using SS-OCT [99].

## 6. Use of AS-OCT in Animal Experiment

**Figure 6:**
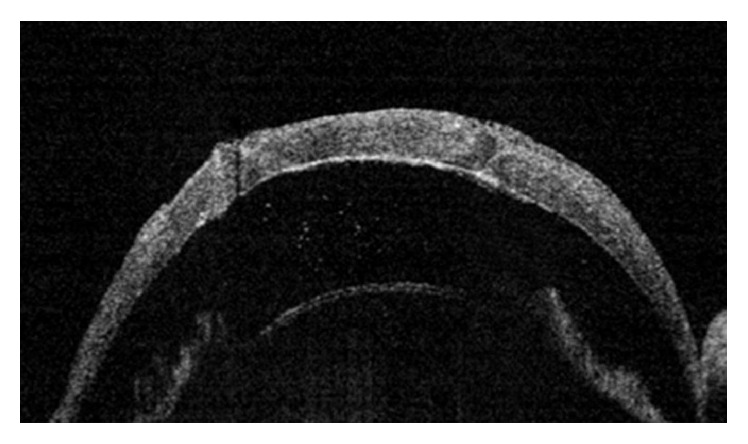
An AS-OCT image demonstrating anterior chamber inflammation in a rejected graft in a rat model.

Experiments using animal models are useful for elucidation of the pathophysiology and development of treatment modalities of corneal and ocular surface disorders. For precise evaluation of changes in anterior segment structures in animal experiments, AS-OCT is advantageous due to its noncontact nature and ability to produce high-resolution images in a short period of time [4]. Han et al. [100] developed a mouse model of corneal endothelial decompensation using transcorneal cryoinjury and proved the persisted corneal edema with AS-OCT. AS-OCT was also useful for visualization of the complications of cryoinjury, including iridocorneal adhesion, AC inflammation, and cataract [100]. They also established an animal model of congenital hereditary endothelial dystrophy using* Slc4a11* knockout (KO) mice and showed the progressive thickening of the cornea of the KO mice using AS-OCT [101]. Using rat penetrating keratoplasty model, Liu et al. [102] recently demonstrated that early increase in AC inflammation and central cornea graft thickness evaluated using AS-OCT was early predictors of graft failure (Figure 6). Because AS-OCT allows the evaluation of the changes in cornea and AC in cases with corneal clouding, it is expected to be particularly useful for animal models of corneal dystrophy or decompensation.

## 7. Conclusion

As a noncontact technique that can produce high-resolution images in a short period of time, AS-OCT is an innovative tool for evaluation of cornea, conjunctiva, sclera, AC, and adjacent anterior segment structure. It has been helpful for diagnosis and management of corneal and anterior segment diseases, planning and performing surgery, monitoring of postoperative course, and researches using human data and animal model. New technologies, such as UHR-OCT or En face OCT, are expected to allow more precise visualization of anterior segment structures, which can improve our ability to diagnosis and treat corneal and ocular surface disorders.
